# 3D Microstructural Architecture of Muscle Attachments in Extant and Fossil Vertebrates Revealed by Synchrotron Microtomography

**DOI:** 10.1371/journal.pone.0056992

**Published:** 2013-02-26

**Authors:** Sophie Sanchez, Vincent Dupret, Paul Tafforeau, Katherine M. Trinajstic, Bettina Ryll, Pierre-Jean Gouttenoire, Lovisa Wretman, Louise Zylberberg, Françoise Peyrin, Per E. Ahlberg

**Affiliations:** 1 European Synchrotron Radiation Facility, Grenoble, France; 2 Department of Organismal Biology, Uppsala University, Uppsala, Sweden; 3 Department of Chemistry, Curtin University, Perth, Australia; 4 Department of Earth and Planetary Sciences, Western Australian Museum, Perth, Australia; 5 Unité mixte de recherche 7193, Centre national de la recherche scientifique, Université Pierre et Marie Curie, Institut des sciences de la Terre de Paris, Paris, France; 6 Unité mixte de recherche 5220, Centre national de la recherche scientifique, Institut national de la santé et de la recherche médicale U1044, Universtité de Lyon, Lyon, France; College of the Holy Cross, United States of America

## Abstract

**Background:**

Firm attachments binding muscles to skeleton are crucial mechanical components of the vertebrate body. These attachments (entheses) are complex three-dimensional structures, containing distinctive arrangements of cells and fibre systems embedded in the bone, which can be modified during ontogeny. Until recently it has only been possible to obtain 2D surface and thin section images of entheses, leaving their 3D histology largely unstudied except by extrapolation from 2D data. Entheses are frequently preserved in fossil bones, but sectioning is inappropriate for rare or unique fossil material.

**Methodology/Principal Findings:**

Here we present the first non-destructive 3D investigation, by propagation phase contrast synchrotron microtomography (PPC-SRµCT), of enthesis histology in extant and fossil vertebrates. We are able to identify entheses in the humerus of the salamander *Desmognathus* from the organization of bone-cell lacunae and extrinsic fibres. Statistical analysis of the lacunae differentiates types of attachments, and the orientation of the fibres, reflect the approximate alignment of the muscle. Similar histological structures, including ontogenetically related pattern changes, are perfectly preserved in two 380 million year old fossil vertebrates, the placoderm *Compagopiscis croucheri* and the sarcopterygian fish *Eusthenopteron foordi*.

**Conclusions/Significance:**

We are able to determine the position of entheses in fossil vertebrates, the approximate orientation of the attached muscles, and aspects of their ontogenetic histories, from PPC-SRµCT data. Sub-micron microtomography thus provides a powerful tool for studying the structure, development, evolution and palaeobiology of muscle attachments.

## Introduction

Muscle attachments, or entheses, on bone are structures of great mechanical significance to the verterate body. They are also important to the science of palaeontology. Vertebrate fossils typically comprise only mineralized hard tissues and thus give a very incomplete picture of the animal. However, if the entheses on the bones can be mapped, this allows the musculature to be at least partly reconstructed - a critical step in the paleobiological interpretation of the fossil. A further level of significance has become apparent in recent years with the recognition that particular muscle attachments can be reliable proxies for cell population identity [1], [2].

In extant vertebrates, most muscle attachments to bone do not leave readily interpretable scars on the bone surface [3]. The traditional approach to reconstructing musculature in fossil vertebrates, which is based on mapping such scars, must thus of necessity produce incomplete and therefore misleading results. However, muscle attachments are also known to modify the underlying cortical bone by embedding extrinsic fibres into the matrix and perturbing the organization of the bone cells [4]. Such histological features can potentially allow not only the position of an enthesis but also the approximate orientation of the muscle (as reflected in the orientation of the attachment fibres) to be identified, even in the absence of surface scarring.

**Figure 1:**
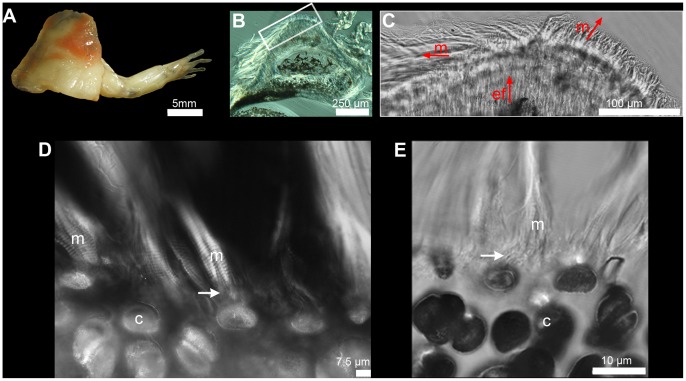
Two-dimensional information on muscle insertions. (A) Dissected forelimb of a *Xenopus tropicalis* specimen. (B–C) Thin section of the humerus after embedment in resin; picture taken under differential interference contrast (DIC). (B) Overview and (C) Details of extrinsic fibres showing that 2D thin sections can only be partially informative about the histology of a muscle attachment: the exact 3D architecture of the extrinsic fibres (ef) remains elusive, in particular in relationship to the fibres of the attaching muscles (m). (D,E) Muscle attachment on the unossified proximal part of an immature humerus. (D) Muscle fibres (m) initially only associate (white arrows) with cells on the surface of this cartilaginous humerus (c) and do not penetrate into the interior of the element. Polarized light. (E) Confocal transmission image in higher resolution showing the fibrous connection between muscle fibres and the outermost cartilage layer (white arrow).

Histological data have traditionally been obtained by sectioning techniques combined with optical microscopy, using either polarized light for the observation of ground-and-polished sections of mineralized specimens, e.g. [5], [6], [7], or paraffin thin sectioning and staining for the observation of demineralized extant specimens (Figure 1), e.g. [7], [8], [9]. All such techniques are destructive and thus unsuitable for rare or unique material. Furthermore, they produce only two-dimensional images of what is fundamentally a three-dimensional tissue organization of fibre lacunae, cell lacunae and growth arrest surfaces. They thus fail to capture 3D data that could illuminate the precise nature of the attachment, the potential orientation of the muscle, and any ontogenetic changes these may have undergone. Among current imaging techniques with limited 3D capability, scanning electron microscopy is only useful for observing surface microstructures on the bone, e.g. [3], [10], while confocal scanning optical microscopy [11] penetrates only a short distance into the bone matrix. Neither is adequate to the task of imaging muscle attachment histology in three dimensions within the complete thickness of the cortical/periosteal bone.

Propagation phase contrast X-ray synchrotron microtomography (PPC-SRµCT) has recently been shown to have great potential for non-destructive, fully 3D visualization of bone microstructures in extant and fossil vertebrates [12]. Here we apply this technique to the study of muscle attachments on the bones of extant and fossil vertebrates. We evaluate the 3D histological architecture as a data source relative to two main criteria. Firstly, in an extant vertebrate with known musculature, does it allow us to identify muscle attachments, distinguish between different types of attachment, and correctly infer the approximate orientation of the muscles? Secondly, can these same features be observed in fossil bones of different types and with different styles of preservation, so as to allow the pattern of muscle attachments, with their histological characteristics and ontogenetic histories, to be reconstructed?

**Figure 2:**
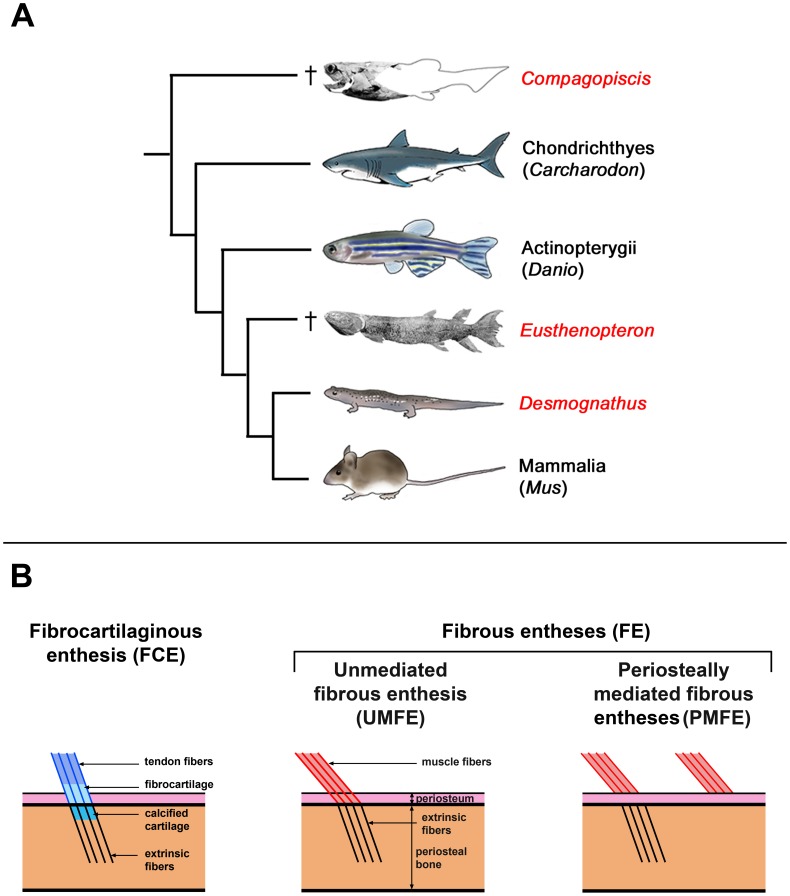
Samples and muscle insertions. (A) Illustrated phylogeny of gnathostomes showing the position and morphology of the studied taxa: *Compagopiscis*, *Eusthenopteron* and *Desmognathus*. (B) Schematic representations of the different types of muscle/tendon attachments. The fibrocartilaginous (FCE) and unmediated fibrous entheses (UMFE) present extrinsic fibres embedded in the bone cortex, while some periosteally mediated fibrous entheses (PMFE) may have no fibre embedded in the bone matrix.

We address the first criterion by examining the relationship between fibres and bone cell organization in different types of muscle attachment in the extant salamander *Desmognathus* (Figure 2A). In order to evaluate the second criterion we apply PPC-SRµCT to fossil bones from the placoderm *Compagopiscis* and the sarcopterygian fish *Eusthenopteron* (Figure 2A). These both date from the Late Devonian Period (approximately 380 million years old) but in other respects provide conveniently contrasting attributes that aid the evaluation of the technique. The *Compagopiscis* bone is a dermal element, the interolateral plate (approximately equivalent to our clavicle), which is believed to have carried hyobranchial muscles [13]. The bone has been completely freed from the surrounding rock by immersion in dilute acetic acid; internal spaces are empty. The *Eusthenopteron* bone is an endoskeletal limb element, the humerus, and has been freed from the rock mechanically; internal spaces are still filled with sediment. The two fossils come from different localities (respectively, Gogo, Western Australia; Miguasha, Québec, Canada) with different geology and styles of preservation. Finally, *Eusthenopteron* is a crown gnathostome closely related to tetrapods (Figure 2A), and might thus be expected to have muscle attachment architecture quite similar to an extant model like *Desmognathus*. *Compagopiscis* on the other hand is a member of the gnathostome stem group (Figure 2A) and thus represents a much deeper, wholly extinct branch in vertebrate phylogeny; the structure of its muscle attachments is accordingly more difficult to predict.

Because muscles attach to the external surfaces of bones, the histological features of an enthesis are always restricted to external appositional bone that has grown by the addition of new matrix to its outer face. In an immature cartilaginous limb element (Figure 1D,E) muscle cells attach to the external surface of the cartilage by means of short fibres. As perichondral and later periosteal bone begins to be deposited between the cartilage and the proximal ends of the muscle cells, gradually separating the two, such fibres may persist, lengthen, and become embedded in the growing bone. By contrast, if the cartilage is later resorbed and replaced by endosteal bone, the latter will not contain any attachment features.

In extant vertebrates, muscular and tendinous entheses can be either fibrocartilaginous (FCE) or fibrous (FE) (Figure 2B) [3], [7], [8], [14], [15]. A FCE comprises four superimposed zones: the most external zone, the tendon, is followed by fibrocartilage, calcified cartilage and finally bone (Figure 2B). Continuous fibres extend from the tendon and fibrocartilage, through the calcified fibrocartilage where they become mineralized, into the underlying, appositionally deposited, cortical bone where they are termed extrinsic fibres [3], [7].

Two types of FE exist (Figure 2B). In a periosteally mediated fibrous enthesis (PMFE), tendon fibres intermingle with and attach to fibres of the periosteum; some of them can be entrapped as extrinsic fibres in the periosteal bone (Figure 2B) [3], [7]. By contrast, in an unmediated fibrous enthesis (UMFE), the tendon fibres always penetrate through the fibrous periosteum and into the periosteal bone (Figure 2B) [3], [7]. The degree of embedment of extrinsic fibres into the periosteal bone thus varies according to the kind of enthesis.

The type of enthesis at a given attachment is thought to be controlled by the epigenetic response to mechanical stresses [16], [17]. Several studies indicate that the density of extrinsic fibres is dependent on the stress exerted on the muscle attachment site [3], [10], [18], [19]. FCE, which usually display a greater density of extrinsic fibres than FE, are able to withstand greater mechanical stresses than FE [16], [17], [20]. If we are able to distinguish different types of entheses in fossil bones we will thus obtain indirect data about the stresses applied to different parts of the musculo-skeletal system in these extinct organisms.

Although several studies have suggested that the density of extrinsic fibres in different categories of muscle attachments may overlap rather than fall into fully discrete clusters, e.g. [10], it seems nevertheless that the two major categories (FCE and FE) can be distinguished by fibre density [3], [7], [10], [19]. However, among the FE, it is difficult to distinguish UMFE from PMFE by observation only of the entrapped extrinsic fibres, e.g. [3]. We use the unique 3D aspect of PPC-SRµCT data to address this difficulty by applying statistical analysis to the spatial organization of bone cell lacunae surrounding the extrinsic fibres.

The results presented here show that 3D bone histology, as revealed by PPC-SRµCT, accurately documents the presence, structure, orientation and ontogenetic history of muscle attachments on the bones of extant and fossil vertebrates. This opens up a major new data source, which allows the relationship between muscle and bone to be investigated more accurately and in greater depth than has been possible with 2D techniques.

## Results

### Establishment of an Extant Model

**Figure 3:**
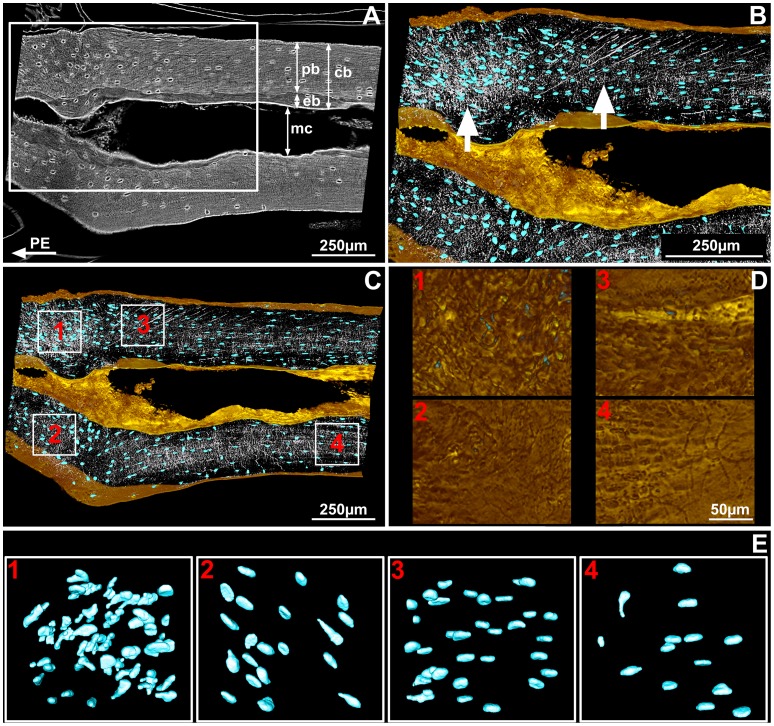
Bone histology of the humerus of the salamander *Desmognathus*. (A) Longitudinal virtual thin section made from scan data (approximately 70 µm thick, voxel size = 0.678 µm) through muscle insertions (located in the white frame). The humerus is oriented with the left side close to the proximal epiphysis (EP) and the right side to the mid-shaft. Abbreviations: cb = cortical bone; eb = endosteal bone; pb = periosteal bone; mc = medullar cavity. (B) Detail of the framed region in Figure 3A, 3D reconstruction of the virtual thin section with osteocytes modelled in blue, extrinsic fibres and canaliculi in white, bone surfaces in gold. The left white arrow shows the unmediated fibrous enthesis (UMFE) and the right white arrow shows the periosteally mediated fibrous enthesis (PMFE). (C) Locations of the four cubes of bone-cell lacunae, on which measurements and statistical tests were performed. (D) Details of bone surface at the location of the four cubes, showing a more rugose surface above cube 1 than above the others. (E) Details of the four cubes after treatment to remove noise and edge-cut lacunae.

*Desmognathus* is a small terrestrial salamander from North America belonging to the family Plethodontidae. The architecture of the humerus, as revealed by our PPC-SRµCT scans, reflects these attributes: it is simply constructed, with an undivided medullary cavity that is lined with endosteal bone (i.e., secondary bone deposit) but lacks trabeculae, and is robustly ossified with a thick cortex. The osteocyte lacunae are large (Figure 3), like in all urodeles [21]. The proximal part of the humerus shows two adjacent areas of muscle attachment containing embedded fibres (Figure 3A–B, white arrows). Both attachments are restricted to the periosteal bone (i.e., cortical primary bone), the fibres terminating abruptly at the contact with the endosteal bone (Figure 3A–B).

**Figure 4:**
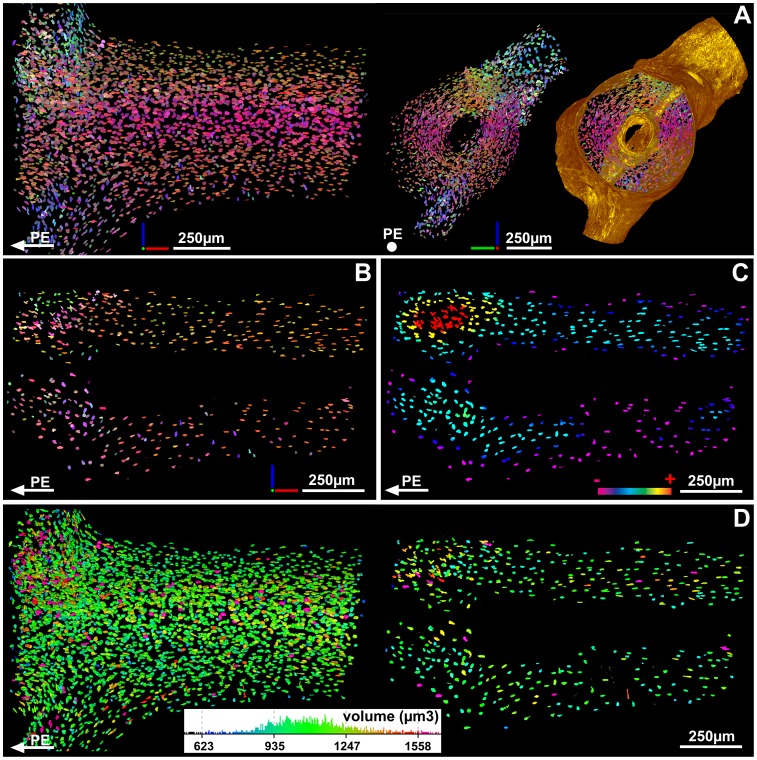
Coloured maps of bone cells in the humerus of the salamander *Desmognathus*. (A) Map of the orientation of the maximum length of each osteocyte in the humerus of *Desmognathus*. Colour coding is represented by a RGB tripod. The orientation of the long bones is given relatively to the proximal epiphysis (PE). The surface of the bone has been added to the right model to visualize the bone-cell orientation in the cortical context. (B) Map of the orientation of the maximum length of each osteocyte lacuna in the humerus of *Desmognathus*, in the same virtual thin section as Figure 3B,C. (C) Map of the density of bone-cell lacunae in the humerus of *Desmognathus* in the same virtual thin section as Figure 3B,C. Colour coding shows the gradation between the densest (+) and least dense (−) regions. (D) Map of the volumes of bone-cell lacunae in the whole humerus of *Desmognathus* (left model) and in the same virtual thin section as Figure 3B,C. (right model). Colour coding is given with the distribution of lacunae volumes within the whole humerus.

The left-hand area, comprising the insertions of some or all of the *dorsalis scapulae*, *procoracohumeralis* and *supracoracoideus* muscles [22], exhibits a rugose surface (Figure 3C–D, cube 1) and contains closely spaced extrinsic fibres. As there is no cartilage layer, it is evidently not a FCE (Figure 2B) despite the surface rugosity, which might suggest such an interpretation. It appears rather to be an UMFE where a tendon attaches to the bone, and the fibres are all incorporated into the bone matrix (Figure 2B) [3], [7], [8]. The osteocyte pattern is visibly disturbed in this area compared to adjacent periosteal bone (Figure 3B–C; Figure 3E; Figure 4B–C; Table 1): the lacunae are closely spaced, forming vertical “stacks” aligned with the fibres. These pattern changes are statistically significant (Text S1). The density of the bone cells is 2.5 times greater than in the rest of the metaphyseal bone where there is no muscle attachment (Figure 4C; Table 1). The volume of the bone cell lacunae is also significantly larger within the area of this enthesis (Figure 4D; Table 1) and almost all the cell lacunae are stellate in shape (Figure 3E).

**Table 1:** Comparison of bone-cell lacuna characteristics in different samples.

taxon	enthesis	density	orientation	volume	star-like shape
***Desmognathus***	UMFE (comparison with region 2)	×2.5	different	different	yes
	PMFE (comparison with region 4)	×1.8	different	similar	no
***Eusthenopteron***	region 1 (comparison with region 4)	×1.5	different	similar	no
	region 2 (comparison with region 4)	×1.5	similar	different*	no
	region 3 (comparison with region 4)	×1.7	different	similar	no
***Compagopiscis***	region 1 (comparison with region 4)	×1.04	different	different	yes
	region 2 (comparison with region 4)	×1.3	different	different	yes
	region 3 (comparison with region 4)	×1.2	different	different	yes

Table summarizing the parameters of bone histology that allow a periosteally mediated fibrous enthesis (PMFE) to be distinguished from an unmediated fibrous enthesis (UMFE) in the humerus of *Desmognathus*. Based on statistically significant results (Text S1), the muscle insertion in the humerus of *Eusthenopteron* can be interpreted as a PMFE and the muscle insertions in the interolateral plate of *Compagopiscis* can be interpreted as UMFE. The *indicates an unexplained increase of the volume of bone-cell lacunae in region 2 in *Eusthenopteron* insertion.

The right-hand attachment (Figure 3B–C) is the origin of the *humeroantebrachialis* muscle [23], [24] and is a PMFE (Figure 2B) [3], [7], where the muscle fibres are bound to the periosteum by a thin layer of connective tissue. It shows neither a distinctive surface texture (Figure 3C–D, cube 3) nor a disturbance of the osteocyte pattern (shape and volume; Figure 3E; Figure 4D; Table 1); it is revealed by the presence of long fibres running obliquely from bottom left to top right through the periosteal bone (Figure 3A–B), by a density of osteocytes 1.8 times greater than in adjacent diaphyseal periosteal bone (Figure 4C; Table 1), and by a slight but statistically significant change of osteocyte orientation (Figure 4A–B; Table 1).

### Descriptions of Fossil Muscle Attachments

#### (a) Eusthenopteron

*Eusthenopteron* is a fish member of the tetrapod stem group, i.e. a close relative of the immediate ancestors of land vertebrates (Figure 2A) [25]. Its paired appendages are fins comprising a basal lobe containing an endoskeleton and a distal fin web supported by dermal lepidotrichia. The endoskeletons are in many respects similar to tetrapod limb skeletons and contain uncontroversial homologues of the tetrapod humerus, femur, radius, ulna, tibia and fibula. The specimen scanned for this paper is a near-complete, fully three-dimensional humerus (lacking only the distal part of the entepicondyle), preserved in articulation with the proximal end of the ulna.

**Figure 5:**
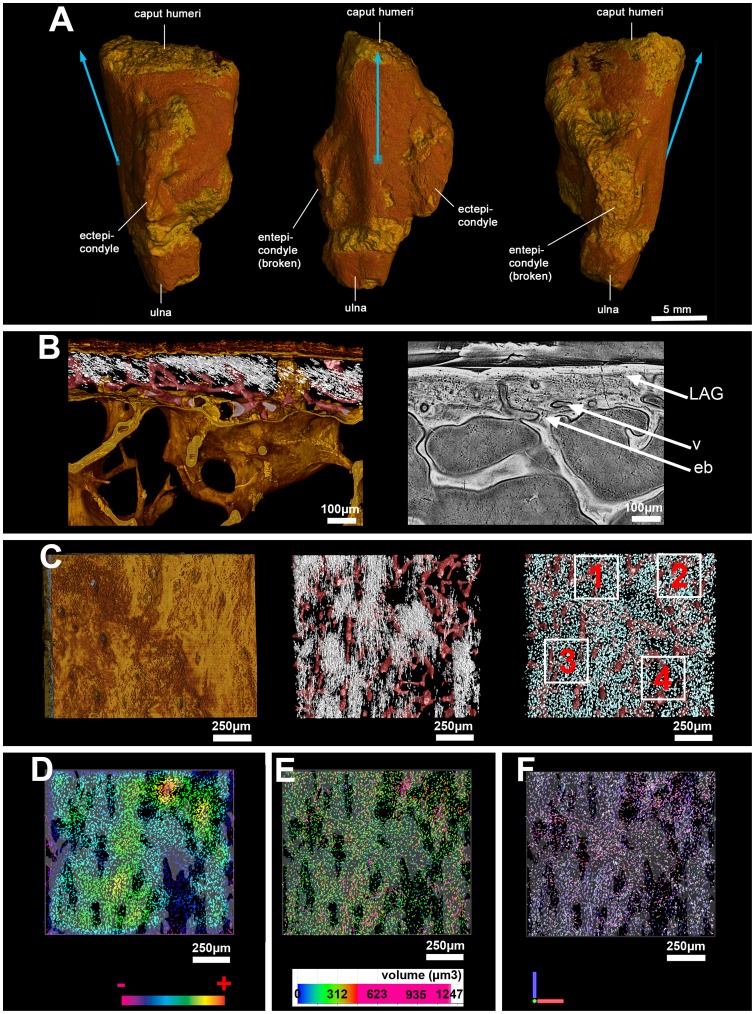
Bone histology of the humerus of *Eusthenopteron*. (A) 3D model of humerus, proximal epiphysis at the top, anatomical articulation with the ulna preserved at the bottom (voxel size = 20.24 µm). The muscle attachment area scanned at 0.678 µm voxel size (Figure 5B–F) is indicated by a blue square with an arrow rising from it that shows the approximate orientation of the muscle. Successive views from left to right: dorsal, mesial and ventral face. (B) Transverse modelled virtual thin section (left) and virtual thin section created from scan images (right) of the high-resolution scan through the muscle attachment area. The proximal end of the humerus is towards the left. The vascular mesh (in pink, v) is surface-parallel and gives off numerous vertical vascular canals that are slightly inclined towards the proximal end of the bone; the fibres (in white) slope obliquely down from proximal to distal. Internally the fibres end at the border of the endosteal bone (eb); externally the great majority do not reach the surface, stopping at the first line of arrested growth (LAG), like most of the vertical vascular canals. (C) Top views showing successively the bone surface of the region of muscle attachment (left), the spatial distribution of the bundles of extrinsic fibres (middle), and the four regions of interest where measurements and statistical tests on bone-cell lacunae were performed (right). The proximal end of the humerus is towards the top in all views (and also in D-F). (D) Top-view map of the density of bone-cell lacunae. In this and the two following images the distribution of the fibres in represented in transparent overlay. (E) Top-view map of the volumes of bone-cell lacunae. (F) Top-view map of the orientation of the maximum lengths of bone-cell lacunae. Same colour codings as for the maps in Figure 3.

In the modelled part of the humerus, which lies on the posterior (or internal) face of the bone immediately dorsal to the longitudinal ridge that divides this face into dorsal and ventral halves (Figure 5A), the deep part of the compact cortex contains hundreds of obliquely oriented extrinsic fibres (Figure 5B). These fibres show a patchy distribution and do not cover the entire area in plan view (Figure 5C). Variations in the pattern of osteocyte lacunae are also noticeable within this area (Figure 5C–F).

**Figure 6:**
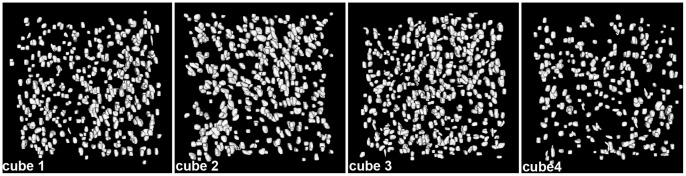
Osteocyte lacunae in the humerus of *Eusthenopteron*. Four cubes of osteocyte lacunae from the humerus of *Eusthenopteron*, in plan view, after treatment to remove noise and edge-cut lacunae.

In order to investigate the possible relationship between presence/absence of fibres and variations in the distribution of bone cells, the osteocyte pattern of four different areas (two fibre-containing areas and two areas of the same size without fibres) within the scanned region was analyzed (Figure 5C). The fibre-containing areas (areas 1 and 3) exhibit a density of bone-cell lacunae (450–500 lacunae) at least 1.5 times greater than in area 4 (Figure 5D; Table 1). The volume of bone-cell lacunae is similar in areas 1, 3 and 4 (Figure 5E; Table 1). Areas 1 and 3 show significant differences in the orientation of bone-cell lacunae compared with region 4 (Figure 5F; Table 1; Text S1). Area 2, which lacks fibres, presents an osteocyte density similar to areas 1 and 3, i.e. 1.5 times greater than in area 4 (Figure 5D; Table 1). The orientation of osteocyte lacunae in area 2 however is similar to the orientation of bone-cell lacunae in area 4 (Figure 5F; Table 1). The volume of bone-cell lacunae in area 2 is significantly greater than in area 4 (Text S1). All the osteocyte lacunae have the same round shape, irrespective of the region they occupy (Figure 6; Table 1).

The majority of the extrinsic fibres terminate externally at an arrested growth surface (Figure 5B–C, LAG) that also forms the termination for most of the blood vessels that extend outwards from the medulla (Figure 5B) [12]. The surface of the bone shows no evidence of muscle insertion at this location of the humerus (Figure 5A–C).

#### (b) Compagopiscis

**Figure 7:**
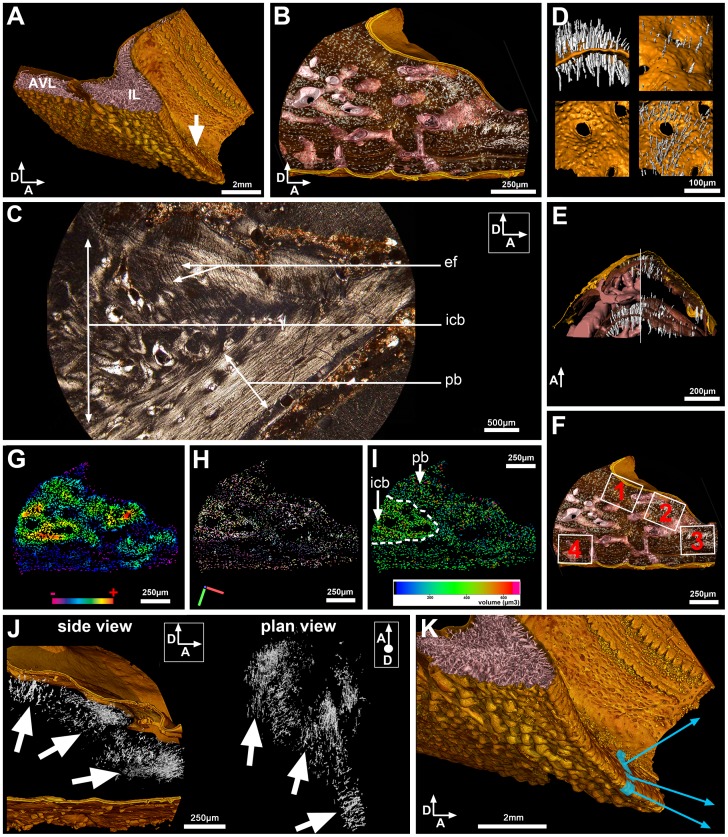
Bone histology of the interolateral plate of the placoderm, *Compagopiscis*. (A) 3D model of interolateral plate (IL) with part of anterior ventrolateral plate (AVL), in anteroventrolateral view (5.05 µm voxel size). Reproduced from [12] with permission. The external (ventral) surface is oriented downwards, anterior to the right. High-resolution scan was done at location of white arrow. (B) Transverse virtual thin section modelled from high-resolution scan (0.678 µm voxel size), showing vascular mesh (pink), bone-cell lacunae (blue), extrinsic fibres (white), lines of arrested growth (brown) and surfaces (gold). Orientation approximately same as (A). (C) Transverse classical thin section through an isolated interolateral of *Compagopiscis* (WAM12.6.03). The white arrows point to a bundle of extrinsic fibres (ef) in the dorsal periosteal bone (pb; approximately corresponding to areas 1 and 2). The periosteal bone surrounds an internal core bone (icb). Picture taken under polarized light. (D) Close-up of growth arrest surface in anteriormost part of interolateral, from a second scan at 0.678 µm voxel size, showing embedded attachment fibres, each associated with a dimple in the surface possibly left by the cell producing the fibre. Clockwise from top left, edge-on view, oblique external view, oblique internal view, oblique internal view without fibres. Holes in the surface are openings for blood vessels. (E) Anteriormost region of interolateral plate showing rows of fibres alternating with vascular layers, same scan as (D). Anterior at top. (F) Virtual thin section (same as B) showing regions where measurements on bone-cell lacunae were performed. (G) Density of bone-cell lacunae in the thin section. (H) Orientation of maximum lengths of bone-cell lacunae. (I) volumes of bone-cell lacunae. Same colour codings as in Figure 4. Note distinctive region of internal core bone on the left-hand side of the section (icb); this tissue is deposited around internal vascular spaces and is never associated with muscle attachments. By contrast, the bone below, to the right of, and above this region (pb) has all been deposited by an external periosteum. (J) 3D model of fibres showing three distinct fibre orientations indicated by the white arrows in side and plan views (orientation of anterior and dorsal indicated with arrows). (K) Interpretative representation of muscle attachments, blue arrows showing approximate orientations of muscles.

*Compagopiscis* is a placoderm, an armoured jawed fish belonging to the gnathostome stem group (Figure 2A). It is known only from the Gogo Formation of Western Australia, famous for its uniquely perfect three-dimensional preservation [26]. Like all placoderms, *Compagopiscis* has a trunk armour or dermal shoulder girdle that forms a complete loop around the body. The anteroventral margin of the shoulder girdle is formed by the interolateral plate, which forms the rear wall of the gill chamber and occupies approximately the same position as our clavicle (Figure 7A). In *Compagopiscis* the dorsal part of the interolateral plate is developed into a denticle-bearing postbranchial lamina that must in life have been covered by the mucosa of the gill chamber wall. Ventral to this lamina is a large forward-facing wedge of bone (Figure 7A, arrow) that has previously been interpreted as an attachment area for hyobranchial musculature, largely on positional criteria [13].

**Figure 8:**
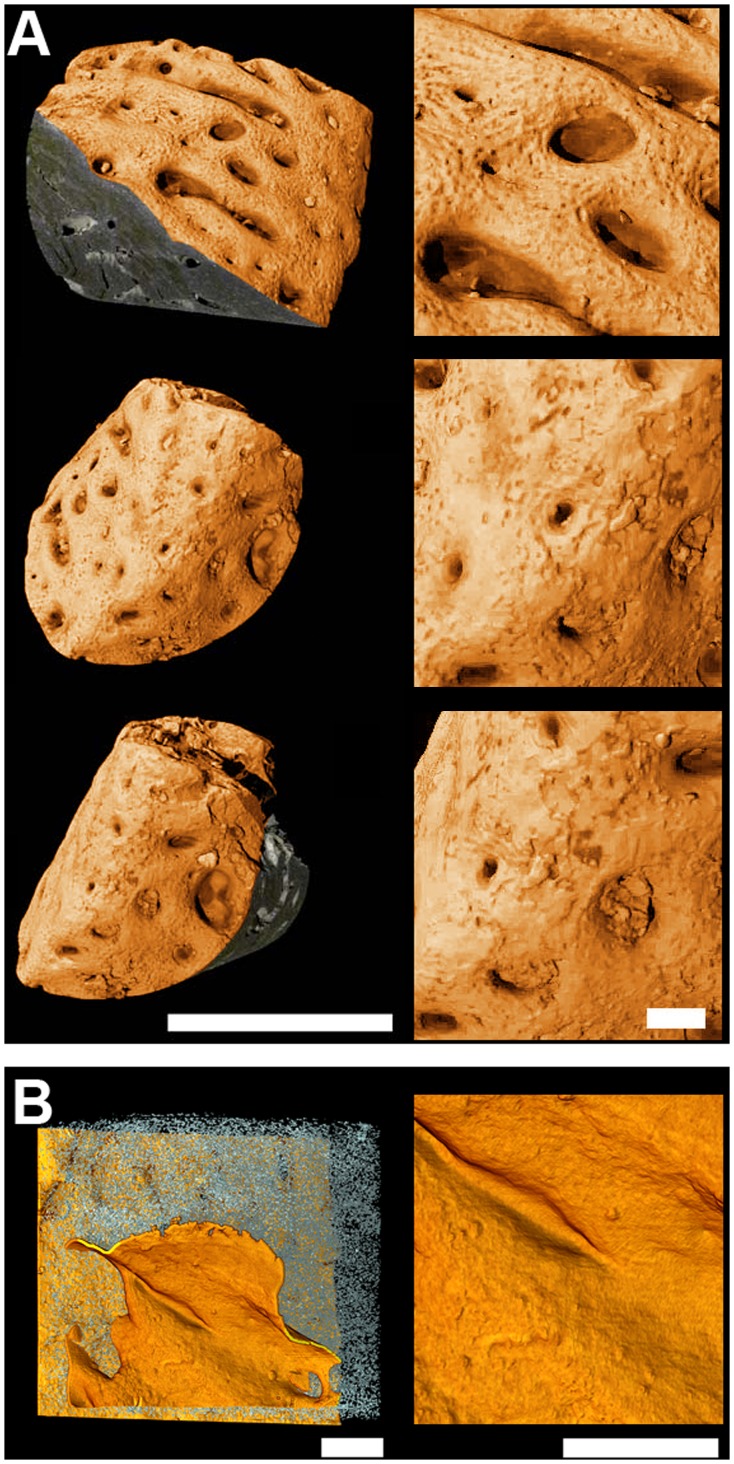
Bone surface of the IL of *Compagopiscis*. (A) On the left, external surface of the anteriormost muscle attachment on the interolateral of *Compagopiscis*, modelled from scan with 0.678 µm voxel size. The bone is oriented obliquely. Top, dorsolateral view; middle, anterior view; bottom, anteroventral view. The dorsal surface shows a dimpled texture identical to that on the arrested growth surfaces in the muscle attachment, whereas the ventral surface is smoother. Scale bar: 1 mm. On the right, close-ups of surfaces showing transition from dimpled (top) to non-dimpled (bottom) surface. The dimples are the size of single cells, and each appears to form the entry point for an attachment fibre that is cemented into the bone. The dimples themselves may have housed cell bodies or reflect delayed mineralisation around the fibres [18]. Scale bar: 100 µm. (B) Smooth external surface of the posteriormost region of muscle attachment. Scale bar: 250 µm.

PPC-SRµCT reveals that the putative attachment area consists of greatly thickened lamellar bone containing large numbers of embedded extrinsic fibres (Figure 7B, C), confirming that the attachment hypothesis is correct. It also contains numerous superimposed arrested growth surfaces, each representing the external surface of the bone at a single instant in time (Figure 7B–E). The surfaces are either simple in shape or very complex, depending on whether or not they capture the growth front in the process of engulfing external surface-parallel blood vessels. In the anterior part of the attachment, these surfaces carry regular dimples, each approximately the size of a single cell and centred on an attachment fibre (Figure 7D); such indentations can also be seen on the external surface of the bone in the same region (Figure 8A). The more posterior part of the dorsal surface of the scanned area shows no obvious dimples (Figure 8B). Within the muscle attachment area, groups of fibres show different alignments. The principal fibre alignments are anteroposterior in the anterior part of the attachment (Figure 7B, E) and two different anterodorsal alignments in the more dorsal part (Figure 7B). Some of the fibres are curved (Figure 7C).

**Figure 9:**
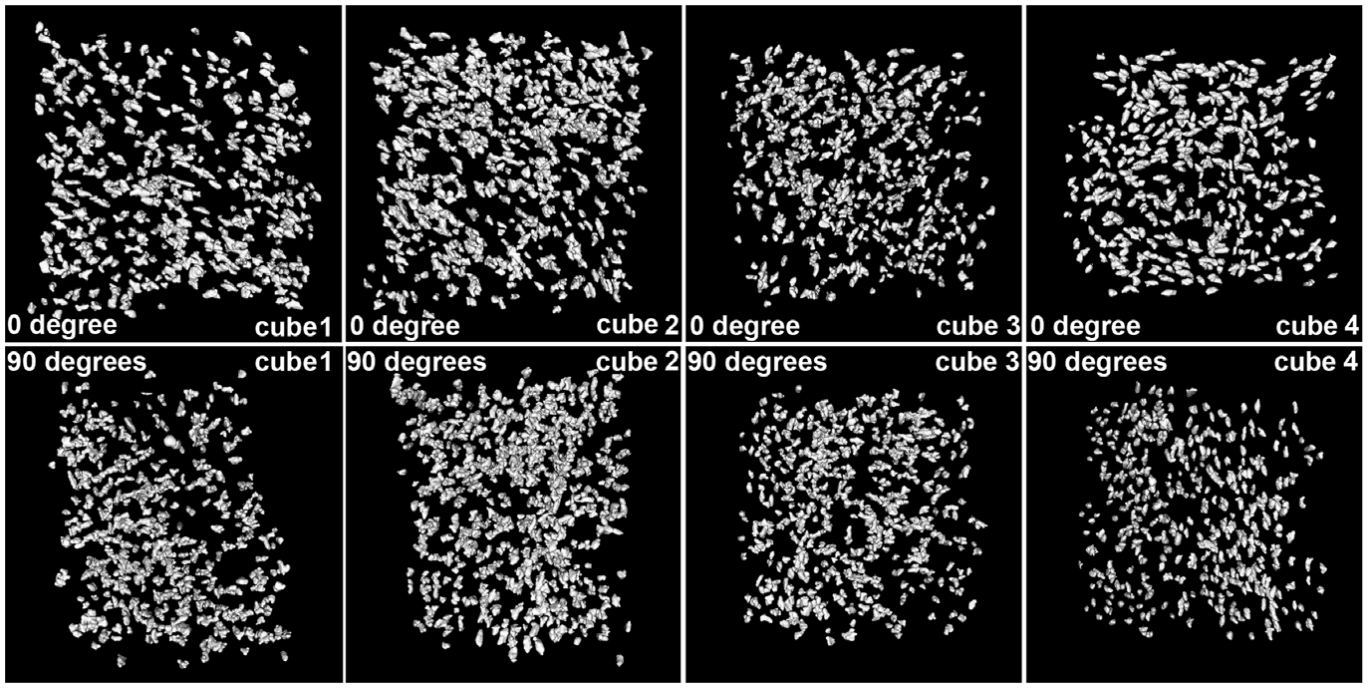
Osteocyte lacunae in the interolateral plate of *Compagopiscis.* Details of the four cubes of osteocyte lacunae in the interolateral plate of *Compagopiscis* after treatment to remove noise and edge-cut lacunae. Top views taken at 0 degrees ( = plan view); bottom views taken at 90 degrees.

The osteocyte pattern has been studied in four different areas of the interolateral plate: in the ventral external region (area 4; Figure 7F), and in three different parts of the muscle attachment - the anterior edge (area 3; Figure 7F), the middle part (area 2; Figure 7F) and the dorsal part (area 1; Figure 7F). The osteocyte density in areas 1, 2 and 3 is slightly higher (1.04–1.3 times greater) than the density in area 4 (Figure 7G; Table 1), and the orientation of their osteocyte lacunae is significantly different (Figure 7H; Table 1; Text S1). All muscle areas (areas 1, 2 and 3) present stellate lacunae that are significantly larger than the oval lacunae in area 4 (Figure 7I; Table 1; Figure 9). The fibres in area 2 do not reach the surface of the bone (Figure 7J). The region anterior to area 3 shows a pattern of successive zones of fibres, which alternate with successive zones of vascular canals parallel to the surface of the plate (Figure 7E; Movie S1). These vascular canals represent vessels that were originally external to the bone but were engulfed by the advancing growth front.

## Discussion

The fact that no calcified cartilage was observed in the entheses either of the extant salamander *Desmognathus* or of the two fossils, even though this tissue fossilizes well and is easy to recognize in a scan section, indicates that all the entheses in our sample material are FE rather than FCE (Figure 2B). The observations of UMFE and PMFE in *Desmognathus* show two significantly different osteocyte patterns (Table 1; Text S1). UMFE contain a greater density of large stellate bone-cell lacunae, differently oriented from the lacunae in surrounding areas. In contrast, PMFE are associated with a density of bone-cell lacunae that is only slightly elevated relative to the surrounding bone; osteocyte lacunae are of the same volume and shape as in the rest of the bone, but their orientation differs slightly (Table 1). Proceeding from the provisional assumption that these patterns also apply to UMFE and PMFE in other vertebrates, we can now interpret the entheses of our fossil taxa.

### Interpretation of Muscle Insertions in Fossils

#### (a) Eusthenopteron

In plan view (Figure 5C), the distribution of fibres in the muscle attachment area on the humerus of *Eusthenopteron* is seen to be markedly heterogenous, with dense patches alternating with gaps. This probably reflects the anatomical organization of the muscle rather than a preservational artefact, because the fibres are either complete or completely absent; we observe no examples of partially preserved fibres, as might be expected if the patchy distribution was due to local variations in preservation. Comparisons of the fibre and osteocyte patterns reveal relationships that probably relate directly to the organization of the muscle attachment, but which are not always easy to interpret because of a paucity of comparable data from other vertebrates. The osteocyte pattern (density, orientation, volume, shape) of the fibre-containing areas (Figure 5C; areas 1 and 3) is similar in every respect to the pattern observed at the location of the periosteally mediated *humeroantebrachialis* enthesis on the humerus of *Desmognathus* (Table 1). In *Eusthenopteron*, the fact that the orientation of bone-cell lacunae in area 2 is similar to that in region 4, and contrasts with the fibre-bearing areas 1 and 3, suggests that there is a strong correlation between the presence/absence of fibres and the orientation of the osteocyte lacunae. The pattern of density distribution is slightly more difficult to interpret. Most of fibre-bearing regions present a high density of osteocytes but the contrary is not necessarily true; area 2 (Figure 5C), which exhibits no fibres, nevertheless shows a high density of bone-cell lacunae similar to the density of fibre-bearing regions. The volume of bone-cell lacunae is definitely not related to the presence/absence of fibres. The highest concentration of big bone-cell lacunae is located in the non fibre-bearing area 2, but the second-highest concentration occurs in a fibre-bearing patch at the right-hand margin of area 3 (Figure 5D).

It is known that muscle attachment areas usually combine entheses of different types [3]. The presence of area 2 in the middle of the fibre-bearing areas suggests that the muscle insertion area may have been composed of a patchwork of different PMFE, some associated with entrapped extrinsic fibres in the periosteal bone (areas 1 and 3; Figure 5C) and others not (area 2; Figure 5C). However, the 2D thin-section data that provide the bulk of published information on muscle attachment architecture are not easy to compare with the 3D data presented here; further studies of recent and fossil muscle attachments by PPC-SRµCT will be needed to establish whether the mosaic organization observed in *Eusthenopteron* is unusual or commonplace.

In addition to this spatial heterogeneity, the muscle attachment also shows a dramatic reorganization through ontogeny. Almost all modelled fibres stop at the deepest visible arrested growth surface in the cortical bone (Figure 5B, C), and as the muscle is unlikely to have simply disappeared at this point in ontogeny, we are forced to conclude that extrinsic fibres abruptly ceased to be embedded into the periosteal bone and afterwards attached only to the periosteum. At the same time, the majority of the vascular canals leading through the bone cortex to the medulla were closed off, indicating that the blood supply to the medulla was drastically reduced [12]. As the density of extrinsic fibres is related to stress constraints [3], we can interpret this combined pattern of vascular capping and cessation of fibre implantation as a reflection of periosteal and biomechanical reorganization in association with abrupt slowing of growth. In vertebrates, it is relatively common to observe the reorganization of extrinsic fibres anchoring soft tissues to the bone during ontogeny. The complexity of this reorganization however has still to be understood. Goodwin and Horner [27] suggested that the reshaping of bundles of extrinsic fibres, connecting the skin to the dome of the dinosaur *Pachycephalosaurus*, could reflect the frequent remodelling of an extremely pliable epidermal layer.

The similarity of the osteocyte pattern in the humerus of *Eusthenopteron* to that in the PMFE of *Desmognathus*, coupled with the abrupt cessation of fibre embedment at a particular point in the ontogenetic trajectory (which implies that the muscle remained attached to the periosteum), allows us to conclude that this enthesis is a PMFE. Like the *humeroantebrachialis* attachment of *Desmognathus*, this muscle insertion is not visible on the surface of the adult bone, and no attachment has been described in this area by previous authors [28]. The detection of its presence demonstrates the superiority of 3D histology over surface scars as a data source for identifying muscle attachments in fossils, and will also be of interest for future biomechanical reconstructions.

In the whole scanned area, the extrinsic fibres are parallel to the long axis of the bone and slope down distally into the cortex; this suggests that the attachment was the insertion of a muscle coming from the internal face of the shoulder girdle (Figure 5A). Given that the insertion lies on the dorsal half of the bone, the muscle was most probably a deep internal member of the elevator group, corresponding to the “premier pronateur” of *Latimeria* [29].

#### (b) Compagopiscis

In the interolateral plate of *Compagopiscis*, the pattern of osteocyte density, orientation, volume and shape of the fibre-containing regions (areas 1, 2 and 3; Figure 7F) is similar in every respect to that observed at the UMFE in the humerus of *Desmognathus* (Table 1). Given that muscle attachments behave similarly in long and flat bones in mammals [16], we thus tentatively interpret the interolateral entheses of *Compagopiscis* as unmediated. The fact that each fibre traverses one or more arrested growth surfaces demonstrates that the fibres were formed externally and became embedded as the bone grew. This conclusion is also supported by the distinctive dimpled texture of the arrested growth surfaces (Figure 7D), particularly in region 3 (Figure 7E, F), where each fibre passes through the centre of a funnel-shaped depression in the surface. This organization suggests that the fibre-generating cells of the FE were interspersed among osteoblasts in a single cell layer on top of the growing bone, so that less bone matrix was generated in the immediate vicinity of the fibres than in the interstices between them. Arrested growth surfaces from non-attachment areas of the bone are not indented in this way.

In region 3 (Figure 7E, F) the fibres reach the external surface of the bone, which is pitted in the same way as the underlying arrested growth surfaces (Figure 8A). However, in regions 1 and 2 (Figure 7F) the external surface of the bone is smooth and the fibres terminate some distance below the surface. This suggests that the entheses in these regions may have changed from UMFE to PMFE during ontogeny. The regular discontinuity of the fibres in region 3 (Figure 7D) suggests that the nature of that enthesis changed cyclically during ontogeny, in association with surface-parallel blood vessels becoming entrapped into the bone.

The fibres are organized in bundles that are differently oriented in regions 1, 2 and 3 (Figure 7F, J), meaning that they actually represent three different entheses. They are probably associated with different muscles: two extending anterodorsally from insertions in the dorsal part of the muscle attachment area and one extending anteriorly from an insertion on the anterior margin of the attachment area (Figure 7K). In agreement with previous authors [13], [30] we can interpret these attachments as representing the insertions for ventral branchial arch and mandibular muscles. The muscle in region 3 (Figure 7E, F) can be tentatively identified as the *coracomandibularis* [13], [31], the muscle in region 2 (Figure 7F) as the *coracohyoideus* [13], [31] and the muscle in region 1 (Figure 7F) as a hypobranchial muscle, the *coracobranchialis* [32]. Some fibres curve in a manner indicating changes in muscle alignment and/or position during ontogeny (Figure 7C, J).

The presence of fibres related to the *coracobranchialis* muscle in region 1 (Figure 7F) conflicts with the hypothesis of Johanson [13], who suggested the presence of a more dorsal musculature, the *clavobranchialis*, and the absence of ventral hypobranchial muscles. The reason for this discrepancy, which has potential phylogenetic significance (*coracobranchialis* muscles are characteristic of chondrichthyans, *clavobranchialis* muscles of osteichthyans), is worth examining more closely because it highlights the value of 3D histology as a data source for the reconstruction of musculature in fossil vertebrates. Johanson [13] used geometrical necessity to infer attachment positions for the *coracomandibularis* and *coracohyoideus* muscles on the shoulder girdle: in order to move the lower jaw and ceratohyal, these anteriorly and anterodorsally oriented muscles must attach near the anterior margin of the ventral part of shoulder girdle. Our discovery of attachment fibres in these areas confirms Johanson’s reconstruction. Johanson could not detect the *coracobranchialis* attachment because it is not visible on the surface, so she concluded that this muscle was absent and that a pocket-shaped attachment area in a more dorsal position housed a *clavobranchialis* attachment. We agree with the existence of this latter attachment area but interpret it instead as possibly housing the ventralmost insertion of the *cucullaris* muscle. Thus, in this instance the data from 3D histology not only ‘fills in the gaps’ between the visible muscle scars: by allowing misattributions to be avoided, it alters homology judgements and affects the whole reconstruction of the branchial musculature.

### Implications for Palaebiological Reconstructions

These results demonstrate that 3D histology is a valuable data source for the study of muscle attachments in both extant animals and fossils. The utility of PPC-SRµCT is currently limited by two main parameters, the field of view of a submicron data set (typically about 2.5 mm in diameter) and the size of sample that can be used for such scanning (a few centimetres). This prevents us from using the technique to produce complete muscle attachment maps of bones. However, within these limitations PPC-SRµCT offers major advantages over conventional 2D approaches. Most of the attachments figured here show no distinctive surface texture and had not been suspected from external observation. PPC-SRµCT thus has the capacity to produce far more complete 3D muscle attachment maps in localized areas than approaches based on the mapping of surface textures. In contrast to sectioning, PPC-SRµCT is non-destructive, meaning that it can be applied without hesitation to rare and valuable fossil material such as the specimens featured here. 3D histology not only reveals the position of the attachment area, but also yields data on the nature of the enthesis, its complexity, the stress this muscle attachment could have endured and the orientation of the muscle. Work is currently in progress to attempt to address the limitations of the technique.

Crucially, PPC-SRµCT is not limited to optimally preserved fossil specimens such as the acid-prepared Gogo Formation fish *Compagopiscis*, with clear internal spaces, but can also obtain 3D data from bones in which the internal spaces are filled with sediment, as exemplified by the humerus of *Eusthenopteron*. Extrinsic muscle attachment fibres are frequently seen in thin sections of fossil bones, e.g. [3], [5], [27], [33], [34], indicating that there is an enormous pool of fossil data that can potentially be retrieved by this technique. Because PPC-SRµCT generates directly comparable data sets from fossil and recent specimens, it provides a unique platform for making in-depth comparisons of muscle attachment architectures across vertebrate phylogeny and deep time. We believe it will greatly aid the investigation of evolutionary change and conservation at the bone-muscle interface.

## Materials and Methods

### Material

This study is based around three model animals: the extant salamander *Desmognathus*, and two Late Devonian (380 million year old) fossil fishes, *Eusthenopteron* from Miguasha, Québec, Canada [28], [35], and *Compagopiscis* from the Gogo Formation, Western Australia [36] (Figure 2A). Additional data were obtained from the extant frog *Xenopus.*

A dried humerus of *Desmognathus* (private collection) with some remaining muscles was used as a test case for imaging muscle attachments in an extant vertebrate with known muscular anatomy. The identification of bone microstructures in a scan data set was carefully checked by comparison with a thin section made at the exact same place after that this bone was scanned [12]. For technical comparative purposes, classical techniques of thin sectioning were also performed on the forelimbs of a specimen of *Xenopus* (Uppsala University livestock) (Figure 1). One forelimb was dried and embedded in a polyester resin; 70 µm thin sections were made. The other forelimb was embedded in a paraffin block and 7 µm thin sections were made and stained with Gabe and Martoja’s trichrome [37]. The thin section shows the ambiguity of interpreting the fibre organization on 2D slices (Figure 1).

A humerus of *Eusthenopteron* (NRM P248d) and an interolateral plate of *Compagopiscis* (BMNH 510007) were selected because they provide a set of contrasting attributes that allow the performance of the technique to be evaluated. The sarcopterygian fish *Eusthenopteron* is a close relative of tetrapods whereas the placoderm *Compagopiscis* is a stem-group gnathostome, belonging to a group with no extant representatives (Figure 2A); the *Eusthenopteron* specimen is endoskeletal bone whereas the *Compagopiscis* specimen is dermal bone; and the *Eusthenopteron* specimen has been mechanically prepared and has internal spaces filled with sediment, whereas the *Compagopiscis* specimen has been acid-prepared and has empty internal spaces.

### Imaging the Sample

Samples were scanned at beamline ID19, European Synchrotron Radiation Facility (ESRF), France. The high-resolution scans presented here all have a voxel size of 0.678 µm; additional lower resolution scans for *Eusthenopteron* (voxel size 20.24 µm) and *Compagopiscis* (voxel size 5.05 µm) are presented as guides to the gross morphology of the bones (Table S1–S2). Most of the scans were made using propagation phase contrast (with monochromatic and pink beam) with a single distance of propagation [12], but some were performed using a holotomographic approach that employs multiple distances and monochromatic beam [38], [39]. When scans were done with a single distance of propagation, a phase retrieval approach based on a homogeneity assumption [40] was employed. All scans were done using a FreLON 2k14 CCD detector and appropriate scintillators (10 µm GGG for high-resolution scans, 125 µm LuAG for scans at 5 µm and a Gadox scintillator of 20 µm thick for 20.24 µm scans). Virtual thin sections were made using the protocol established by Tafforeau and Smith [41] for virtual histology of teeth. The segmentation of the scan data sets was done using the software VGStudio MAX version 2.1 (Volume Graphics Inc., Germany).

### Statistics

In order to discriminate the different types of entheses, statistical tests were performed on virtual 3D cubes of bone-cell lacunae, of identical size within each taxon and extracted from precise locations. These cubes were created with VGStudio MAX version 2.1 (Volume Graphics Inc., Germany). The quantification was based on in-house developed softwares (Text S1). To perform these tests, we used the open-access statistical package R version 2.14.2 (http://www.r-project.org/) and its interface Rcmdr. A Mann-Whitney test was used to test the differences of volume between ostecytic lacunae. A Chi^2^ test was used to verify the (in)homogeneity between the orientations of maximum-length of osteocytic lacunae (Text S1). The mapping visualization of the volume of the bone cells is quantitative and was done using the Defect Detection Option in VGStudio MAX version 2.2 (Volume Graphics Inc., Germany). The visualization of the density mapping is only qualitative and was made possible thanks to a Gaussian blur filter in Photoshop CS4 version 11.0.2 (Adobe Inc.). The visualization of the orientation of the bone cells is only qualitative and is based on in-house developed software.

### Ethics Statement

The forelimb of *Desmognathus quadramaculatus* comes from one specimen collected under ethical guidelines for research study by Bruce et al. [42]. All procedures described within regarding the material of *Xenopus tropicalis* were approved by the Uppsala Local Ethics Committee for animal care and use, and were performed in accordance with guiding principles for the care of laboratory animals.

## Supporting Information

Figure S1 Virtual thin section showing the locations in the humerus of *Desmognathus* where the cubes of osteocyte lacunae were extracted. (Figures S1–S4 and Tables S3–S7 relate to Text S1.)
(TIF)

Figure S2 Comparative series from cube 3 shown in Figure S1. These series show the organization of bone cell lacunae after the successive use of two filters at 0 degrees, 90 degrees and 45 degrees with an oblique inclination of 45 degrees downwards. (A) Series illustrating the raw data with segmentation noise indicated by red arrow. (B) Series illustrating the action of the first filter: the segmentation noise has disappeared. The yellow arrow shows a cut osteocyte lacuna at the edge of the cube. (C) Series illustrating the action of the second filter: all the osteocyte lacunae that were cut at the edges have disappeared.
(TIF)

Figure S3 Test of the normality of the distribution of bone cell lacuna volumes in cube 3 from *Desmognathus*. (A) Measurements of volumes of bone cell lacunae. (B) Qqplot showing that the distribution is not normal.
(TIF)

Figure S4 Box plots showing the distributions of bone cell-lacuna volumes in cubes 3 and 4 from *Desmognathus*. A Mann Whitney test shows a significant difference of volumes between the osteocyte lacunae from the two cubes (within 95% confidence limits).
(TIF)

Table S1 Acquisition parameters for scans done with a monochromatic beam.
(XLS)

Table S2 Acquisition parameters for scans done with a pink beam.
(XLS)

Table S3 Counts of osteocyte lacunae in the four cubes extracted from the humerus of *Desmognathus.*
(XLSX)

Table S4 Counts of osteocyte lacunae in cubes 3 and 4 from *Desmognathus* whose maximum length coincides with one referential axis: X, Y or Z.
(XLSX)

Table S5 Number of bone cell lacunae in the sample cubes of *Desmognathus*, *Eusthenopteron* and *Compagopiscis*.
(XLSX)

Table S6 Orientation of the maximum length of the bone cell lacunae in the sample cubes of *Desmognathus*, *Eusthenopteron* and *Compagopiscis*.
(XLSX)

Table S7 Statistical analysis of the volume of the bone cell lacunae in the sample cubes of *Desmognathus*, *Eusthenopteron* and *Compagopiscis*. (Because the tables of raw data of lacuna volumes are very large, we present here only the statistical results. The complete data set can be obtained from the corresponding author on request).
(XLSX)

Text S1 Supporting information on the statistical methods used to analyse the distribution and characteristics of bone cell lacunae.
(DOCX)

Movie S1 3D model of the anterior tip of the interolateral plate of *Compagopiscis* showing the alternating organization of the extrinsic fibres and the vascular mesh. The bone surface is in gold, the vascular canals in pink, the successive surfaces of arrested growth in brown and the extrinsic fibres in white.
(AVI)
