# Supplementary figures and images for: 3D Microstructural Architecture of Muscle Attachments in Extant and Fossil Vertebrates Revealed by Synchrotron Microtomography
Source: PLoS One. 2013 Feb 26;8(2):e56992. doi: 10.1371/journal.pone.0056992 (PMC3582629; doi:10.1371/journal.pone.0056992)

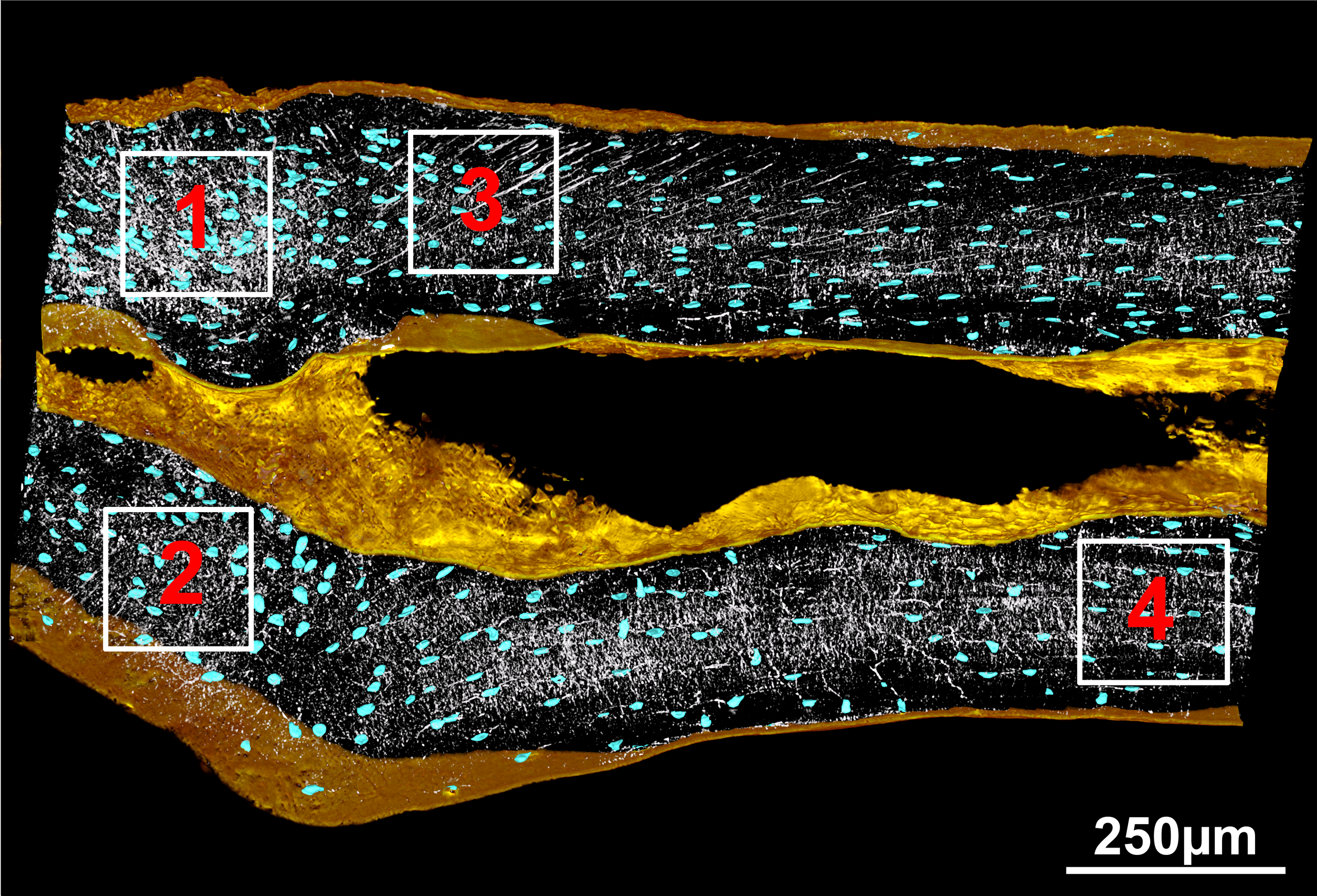

Supplement: Figure S1 — Virtual thin section showing the locations in the humerus of Desmognathus where the cubes of osteocyte lacunae were extracted. (Figures S1–S4 and Tables S3–S7 relate to Text S1.) (TIF) [file pone.0056992.s001.tif]

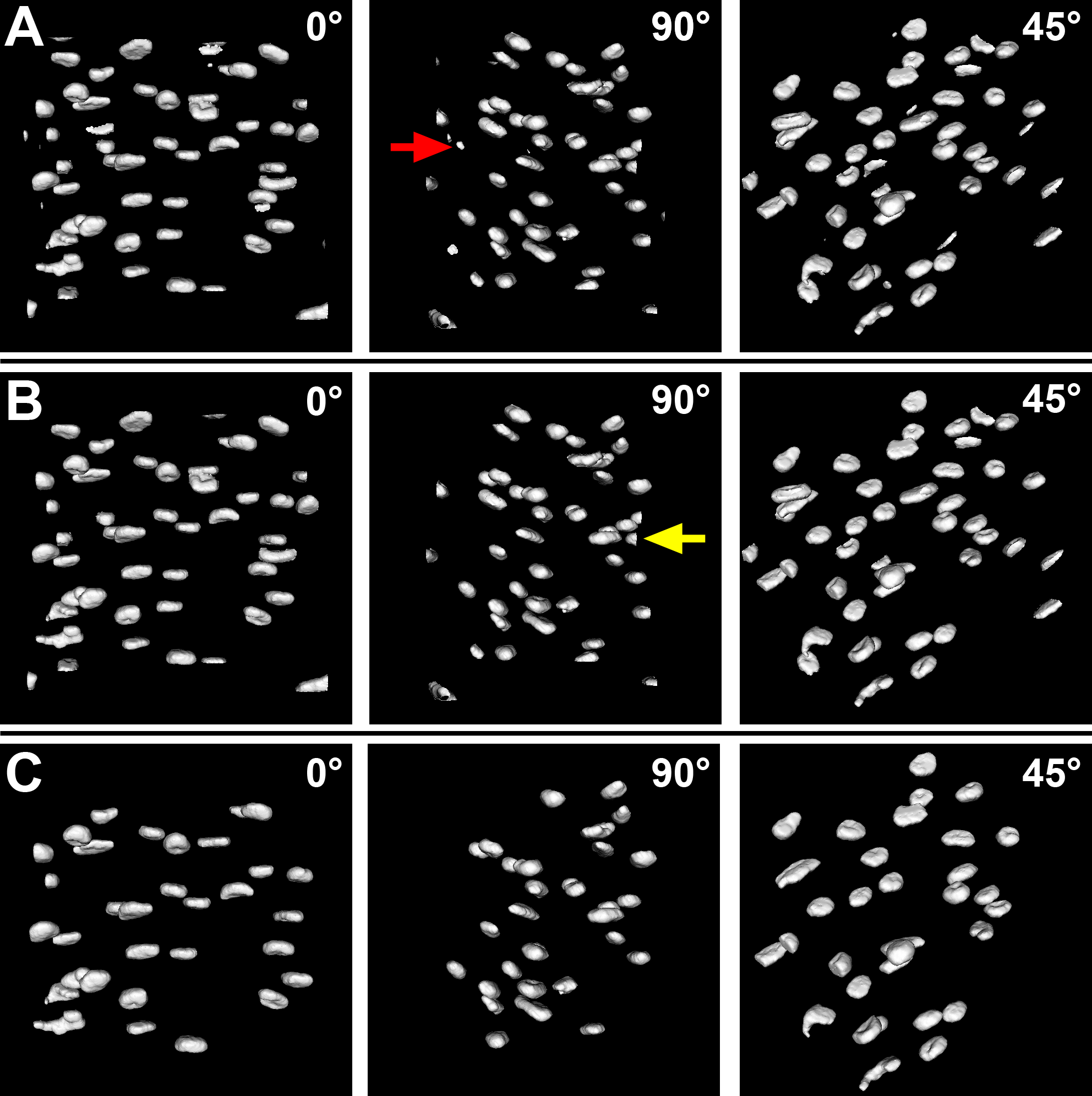

Supplement: Figure S2 — Comparative series from cube 3 shown in Figure S1. These series show the organization of bone cell lacunae after the successive use of two filters at 0 degrees, 90 degrees and 45 degrees with an oblique inclination of 45 degrees downwards. (A) Series illustrating the raw data with segmentation noise indicated by red arrow. (B) Series illustrating the action of the first filter: the segmentation noise has disappeared. The yellow arrow shows a cut osteocyte lacuna at the edge of the cube. (C) Series illustrating the action of the second filter: all the osteocyte lacunae that were cut at the edges have disappeared. (TIF) [file pone.0056992.s002.tif]

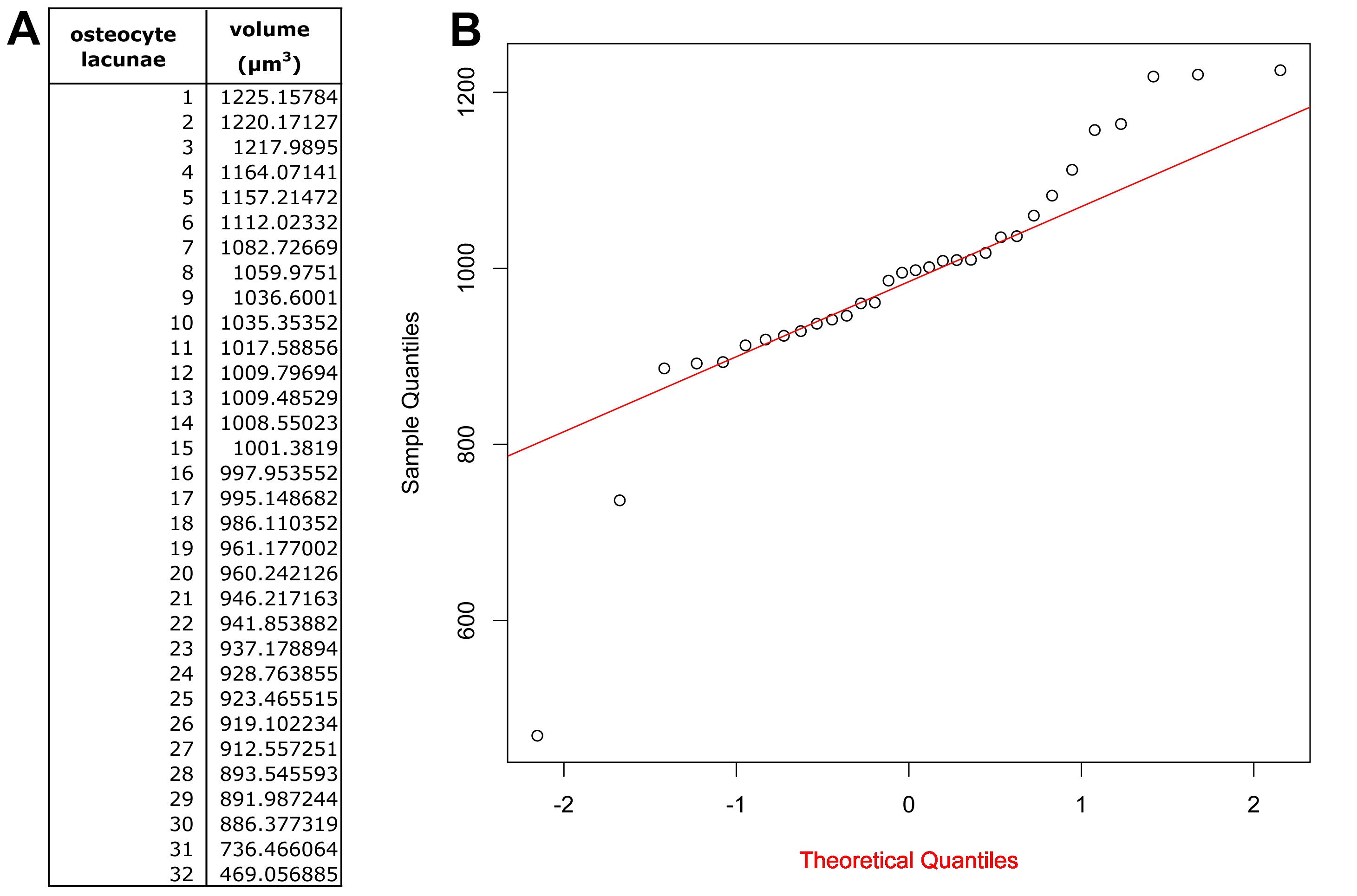

Supplement: Figure S3 — Test of the normality of the distribution of bone cell lacuna volumes in cube 3 from Desmognathus. (A) Measurements of volumes of bone cell lacunae. (B) Qqplot showing that the distribution is not normal. (TIF) [file pone.0056992.s003.tif]

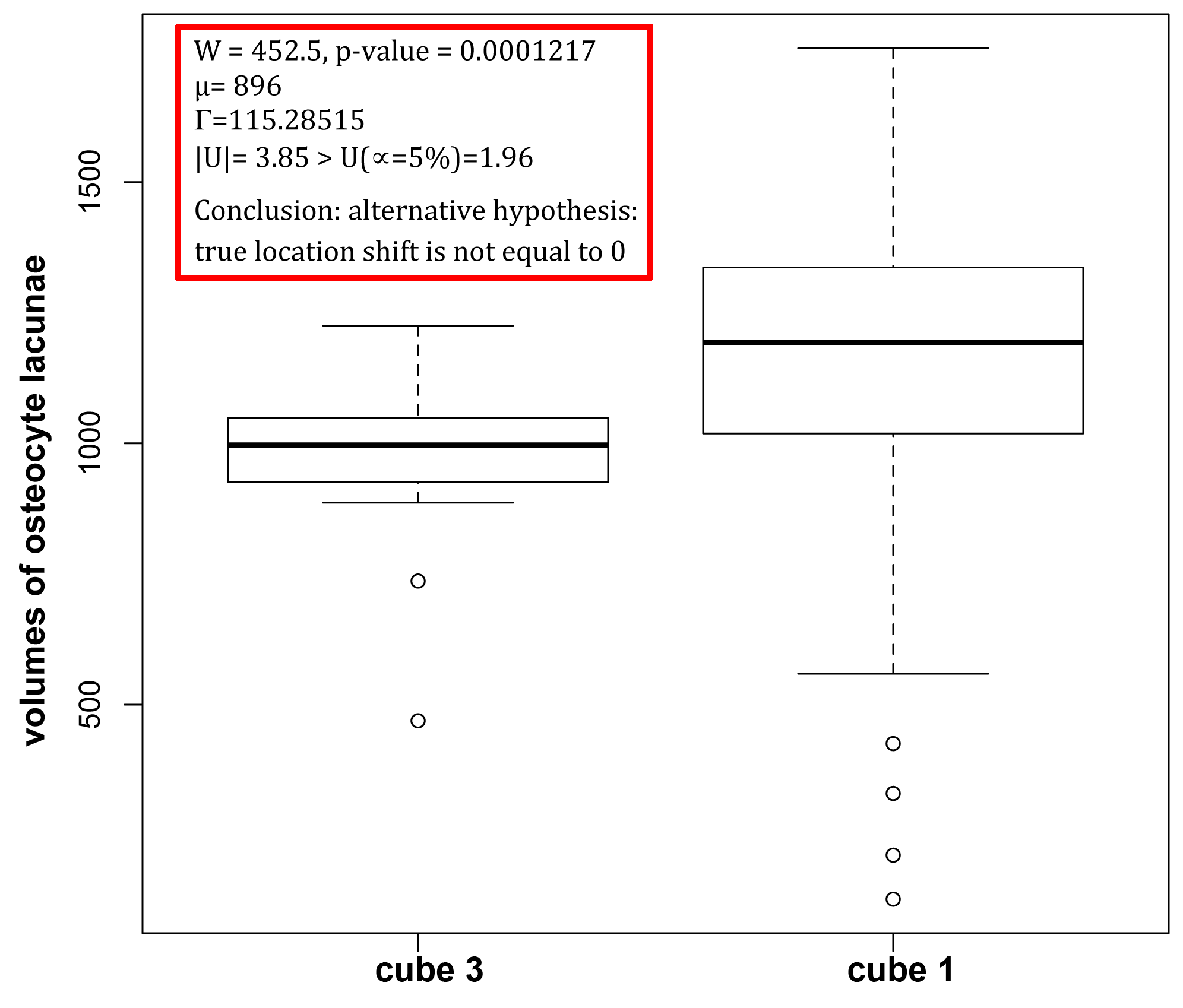

Supplement: Figure S4 — Box plots showing the distributions of bone cell-lacuna volumes in cubes 3 and 4 from Desmognathus. A Mann Whitney test shows a significant difference of volumes between the osteocyte lacunae from the two cubes (within 95% confidence limits). (TIF) [file pone.0056992.s004.tif]
