# Supplementary material for: 3D Microstructural Architecture of Muscle Attachments in Extant and Fossil Vertebrates Revealed by Synchrotron Microtomography
Source: PLoS One. 2013 Feb 26;8(2):e56992. doi: 10.1371/journal.pone.0056992 (PMC3582629; doi:10.1371/journal.pone.0056992)
Supplement: Text S1 — Supporting information on the statistical methods used to analyse the distribution and characteristics of bone cell lacunae. (DOCX) [file pone.0056992.s012.docx]

**Supporting information on the statistical method**

**Data collection**

Cubes of osteocyte lacunae of given dimensions were extracted virtually from regions of interest in the bones of *Desmognathus*, *Esuthenopteron* and *Compagopiscis*. In order to illustrate the methodological process, we use here the example of the humerus of *Desmognathus* (Figure S1).

For quantification purposes, each cube was first segmented using the image processing software VGStudioMax version 2.1 (Volume Graphics Inc., Germany), then extracted and rendered as a binary volume using the open-access software ImageJ64 version 1.43r (http://rsb.info.nih.gov/ij). The remainder of the process was performed using in-house developed software. The segmentation noise was filtered by applying a connected component analysis algorithm, which labels each connected region. Each labelled component with a volume smaller than a given threshold, and thus corresponding to segmentation noise, was removed. Similarly, components with a volume larger than a second threshold and likely to correspond to other structures than osteocyte lacunae were also eliminated (Figure S2A-B). The number of osteocyte lacunae per cube, taken as an indicative reference of the density of bone cell lacunae, was counted at this stage (Table S3).

Because the extraction of the cubes involved cutting some osteocyte lacunae, a second filter was applied which enabled the removal of those osteocytes that were cut at the edges (Figure S2C).

After this stage, the volume and orientation of each labeled osteocyte lacunae was calculated. The orientation was defined as the main direction of the best ellipsoid fitting the osteocyte lacunae. This stage allowed the quantification of the mean volume and orientation distribution within each cube. The parameters corresponding to each lacuna were listed in an output file (e.g., Figure S3A). For each parameter, it was then possible to perform a statistical analysis.

**Statistics applied for the comparison of bone-cell lacuna volumes:**

The volume of each osteocyte lacuna was measured and listed (Figure S3A). The distribution of each sample was checked using the graphical technique of quantile-quantile plot (‘qqnorm’ command in R). The results revealed that all distributions were not normal (Figure S3B). Because the samples are independent and the number of osteocyte lacunae is different according to the samples, we performed a Mann-Whitney test in order to verify if the volume of ostecyte lacunae was significantly different in areas of muscle/tendon attachments (‘wilcox.test’ command with the interface R Commander) (Figure S4).

**Statistics applied for the comparison of bone-cell lacuna orientations:**

The coordinates X,Y,Z of the main orientation vector corresponding to the axis of maximum length of each osteocyte were calculated. The 3D X-Y-Z referential is identically oriented in all cubes. The axes of maximum lengths were reported in a table (Table S4).

Because the samples are independent and the variables qualitative, we performed a Chi^2^ test in order to verify the (in)homogeneity between the distributions of cubes from areas of muscle/tendon attachments and control areas (‘chisq.test’ command with R). Here we illustrate the results of the Chi^2^ test on the cubes 3 and 4: Chi^2^=6.761, degree of freedom = 2. Chi^2^=6.761> theoretical Chi^2^=5.99 for ∝=5%. We can therefore conclude that the orientations between the osteocyte lacunae from the cubes 3 and 4 are significantly different (within 95% confidence limits).

**Raw data and statistical results**

The raw data and statistical results are summarized in Tables S5-S7.
